# Targeting the non-classical estrogen pathway in neurodegenerative diseases and brain injury disorders

**DOI:** 10.3389/fendo.2022.999236

**Published:** 2022-09-15

**Authors:** Zsombor Koszegi, Rachel Y. Cheong

**Affiliations:** ^1^ Institute of Metabolism and Systems Research, University of Birmingham, Birmingham, United Kingdom; ^2^ Timeline Bioresearch AB, Medicon Village, Lund, Sweden

**Keywords:** estrogen, non-classical, non-genomic, neurodegeneration, neuroprotection

## Abstract

Estrogens can alter the biology of various tissues and organs, including the brain, and thus play an essential role in modulating homeostasis. Despite its traditional role in reproduction, it is now accepted that estrogen and its analogues can exert neuroprotective effects. Several studies have shown the beneficial effects of estrogen in ameliorating and delaying the progression of neurodegenerative diseases, including Alzheimer’s and Parkinson’s disease and various forms of brain injury disorders. While the classical effects of estrogen through intracellular receptors are more established, the impact of the non-classical pathway through receptors located at the plasma membrane as well as the rapid stimulation of intracellular signaling cascades are still under active research. Moreover, it has been suggested that the non-classical estrogen pathway plays a crucial role in neuroprotection in various brain areas. In this mini-review, we will discuss the use of compounds targeting the non-classical estrogen pathway in their potential use as treatment in neurodegenerative diseases and brain injury disorders.

## Introduction

Estrogens are a group of gonadal sex hormones that exist naturally in three different forms in humans. 17β-estradiol (E2) is the most dominant biological form, followed by estrone (E1) the intermediate form, and estriol (E3), which has very low levels in the body that are only increased during pregnancy. In this mini-review, we will use the abbreviation E2 to refer to 17β-estradiol and will focus predominantly on this form as this is the most abundant and most of the research has been largely focused on studying this molecule. In addition to its role in reproductive functions, E2 has a profound influence on the central nervous system (1, 2). This has contributed to the interest generated around the impact of E2 on neuronal function in health and disease. Investigations over the past few decades have shown that E2 has the potential to prevent or counterbalance the symptoms of neurodegenerative diseases. The gender differences observed in two of the most common neurodegenerative diseases, Alzheimer’s disease (AD) and Parkinson’s disease (PD), clearly suggest this role (3–5). Although there is no conclusive evidence for E2 treatment in neurodegenerative diseases in human clinical trials, there have been several *in vivo* rodent and *in vitro* cell line models that indicate the therapeutic effects of E2. This mini-review will discuss the neuroprotective, non-classical effects of E2 in the context of some of the most typical neurodegenerative cases (that is AD and PD) as well as brain injuries that possibly lead to neurodegeneration (traumatic brain injury and stroke) and highlight the use of some of the non-classical E2 analogues to potentially prevent or treat these disorders.

### Classical versus non-classical estrogen pathways

E2 regulates cellular processes by binding to specific estrogen receptors (ERs) with two distinct modes of action, broadly classified as the classical and non-classical estrogen pathway. Stimulation of the classical pathway results in direct transcriptional effects through the binding of E2 to its intracellular receptors (ERα and ERβ) and activation of the estrogen response element (ERE) (6). In contrast, the non-classical pathway involves the rapid activation of ion channels and intracellular second messenger signaling pathways. The latter is followed by the stimulation of an array of gene transcription factors, but activation *via* the non-classical pathway is ERE-independent. The non-classical pathway is often described as rapid, as the activation of intracellular signaling pathways can be detected in a matter of seconds, as first demonstrated by Szego and Davis, whereby E2 induced an increase in cyclic adenosine monophosphate (cAMP) levels in the uterus few seconds following administration (7). However, this rapid signaling pathway activation will also often lead to gene transcription, which can be detected at a slower rate. One of the most important transcription factors of the non-classical pathway is the cAMP response element-binding protein (CREB), which has been implicated in multiple studies (8–10).

**Figure 1:**
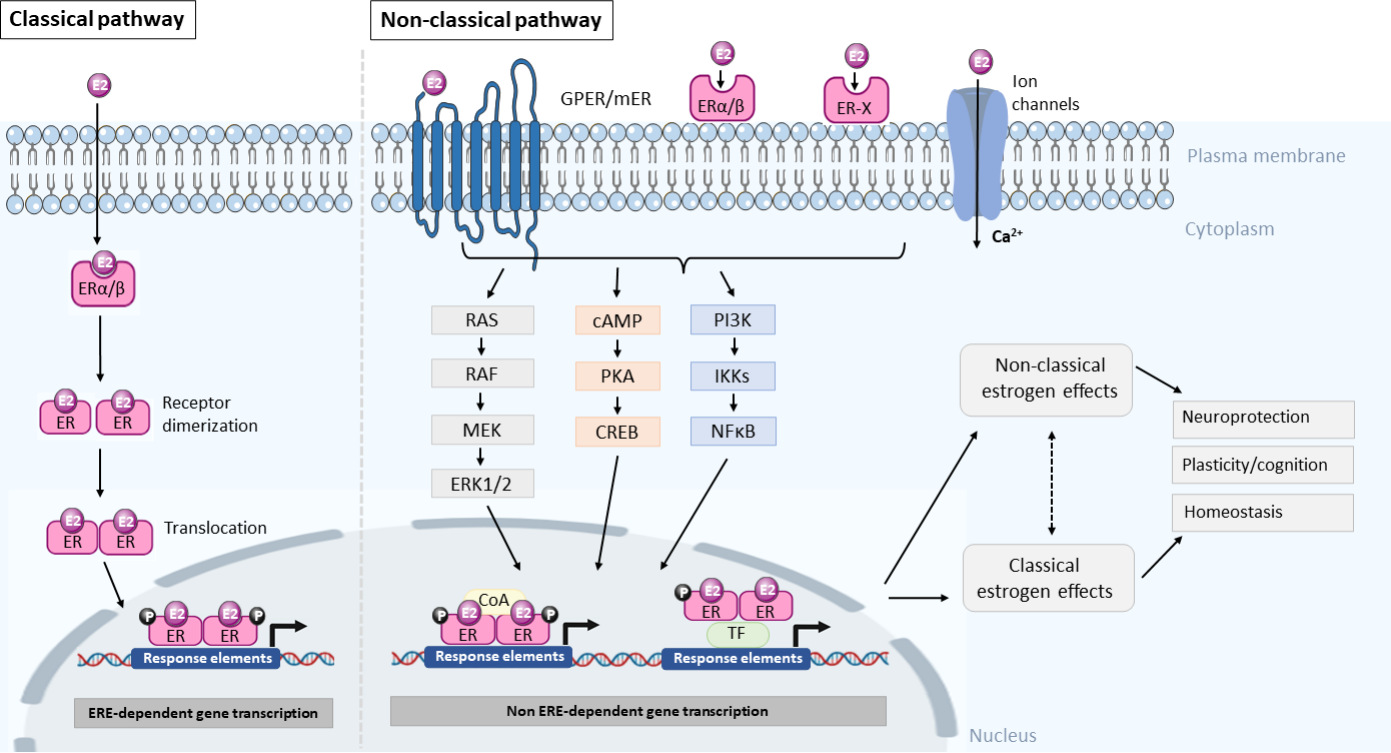
Summary diagram of the classical and non-classical modes of estrogen action. In the classical pathway, E2 crosses the plasma membrane by diffusion and binds to the estrogen receptor (ER) and forms an E2-receptor complex, which dimerizes and translocates to the nucleus to regulate gene transcription through an estrogen response element (ERE) dependent manner. In the non-classical pathway, E2 interacts with membrane bound estrogen receptors (mER), G-protein coupled estrogen receptors (GPER), ER-X, or classic ER (ERα/β) and activates kinases and second messenger signaling pathways to phosphorylate transcription factors (TF) or coactivators to influence gene transcription in the nucleus via a non-ERE-dependent manner. The resultant effect of activating these pathways is neuroprotection, modulating plasticity and cognition as well as maintenance of homeostasis. However, the extent to which the non-classical and classical pathways crosstalk or interact with each other is not known. It is likely that both pathways contribute to neuroprotection and homeostasis. RAS, Ras small GTPase, RAF, Raf kinase, MEK, mitogen-activated protein kinase, ERK1/2, extracellular signal-regulated kinase 1/2, cAMP, cyclic adenosine monophosphate, PKA, protein kinase A, CREB, cAMP-responsive element-binding protein, PI3K, phosphatidylinositol-3 kinase, IKKs, IκB kinases, NFκB, nuclear factor kappa-light-chain-enhancer of activated B cells, coA, coactivator.

Apart from the classical ERα and ERβ, experiments looking at the rapid signaling pathway activation by E2 highlighted that these classical intracellular receptors – mediating ERE-dependent gene transcription – might not be sufficient to account for the variety of responses observed. This led to the discovery of membrane linked receptors, which can be membrane-localized classical ERα and ERβ or other types, for example, the ER-X and the G protein coupled GPR30 (GPER1) (11–13), which are all different from the classical receptors in their structure, localization, as well as modes of action. A schematic illustration of the classical and non-classical modes of E2 action is depicted in **Figure 1**.

### Mechanism for non-classical E2 neuroprotection

There are several possible molecular mechanisms contributing to non-classical E2 neuroprotection, such as control of neuroinflammation, myelin protection, mitochondrial protection and control of oxidative stress, regulating autophagy as well as maintenance of synaptic transmission and plasticity. One of the important protective actions of E2 is in the control of neuroinflammation whereby E2 reduces the secretion of proinflammatory cytokines and interleukins and thereby reducing microglia activation *via* the inhibition of the nuclear factor kappa-light-chain-enhancer of activated B cells (NFκB) signaling pathway (14, 15). In addition, the neuroprotective effects of E2 are in part due to its protective actions on myelin and remyelination, which can be mediated by activation of the phosphoinositide 3-kinases (PI3K)/protein kinase B (Akt)/mammalian target of rapamycin (mTOR) signaling pathway (16–18). Dysfunction in the myelin sheaths is often a common feature in neurodegenerative diseases such as AD and PD as well as in other central nervous system pathologies, such as traumatic brain injury (TBI), stroke and multiple sclerosis. In these neuropathological conditions, E2 has been shown to upregulate genes involved in synaptogenesis, axonal repair and synaptic plasticity, such as Bcl2, TrkB and cadherin-2 (19–21). Another way in which E2 exerts its neuroprotective effects is against oxidative stress through the protection of mitochondrial function and by reducing the production of reactive oxygen species (22, 23). Under pathological conditions, E2 may also elicit various of the above-mentioned responses, but may also promote the release of different neurotrophic factors such as the glial cell line-derived neurotrophic factor (GDNF), insulin-like growth factor 1 (IGF-1) and brain-derived neurotrophic factor (BDNF) to protect neurons and promote reparation of injured neuronal circuits (24, 25).

### Compounds targeting the non-classical estrogen pathway

Importantly, previous findings indicate that apart from the classical estrogen pathway, the non-classical pathway also plays a role in ameliorating neurodegeneration in disease models. The latter is of particular interest as E2 replacement therapy, which affects both the classical and non-classical pathways, has been shown to not only increase the risk of myocardial infarction or coronary heart disease but could potentially lead to an array of side effects, including increased risk of breast cancer and stroke (26–28). Therefore, there has been a renewed interest in developing new compounds that are able to trigger protective or restorative effects without the risk of unwanted side effects. One of these groups of such compounds is the ‘selective estrogen-receptor modulators’ (SERMs), which are non-steroidal molecules with specific mechanism of action in target tissues. They primarily act as partial ER agonists in the target tissue while being antagonists in non-target tissues. Some SERMs, for example, tamoxifen and raloxifene are already in clinical use for pre- and post-menopausal women (29), while others, such as the GPER1 agonist G-1 or the STX (a Gq-coupled membrane ER agonist) are used in preclinical animal studies (30, 31). The challenge with SERMs lies in the balance between the efficacy of the agonistic profile and, at the same time, the reduction of unwanted side effects on non-target tissues. While newer third generation SERMs, such as bazedoxifene, ospemifene and lasofoxifene, have improved efficacy, their use as SERMs in the brain is not known (32). Other important compounds are the ‘activators of non-genomic estrogen-like signaling’ (ANGELS), which is a novel group in E2 therapy that is aimed at targeting the non-classical E2 pathway. Three of these molecules are known, estren (4-estren-3alpha, 17beta-diol), compound A, and compound B, which are all capable of triggering the non-classical E2 pathway (33, 34). However, these compounds are yet to be used in clinical practice, although estren has been found to have protective effects on basal forebrain cholinergic neurons (35, 36), indicating that there is prospect for the use of these non-classical activators as treatment for neurodegenerative diseases.

## Alzheimer’s disease

### Pathophysiology

Alzheimer’s disease (AD) is a chronic progressive neurodegenerative disorder, characterized by distinct hallmark pathologies, such as the presence of amyloid plaques, which comprises primarily of aggregated amyloid β (Aβ) peptide, and formation of neurofibrillary tangles with hyperphosphorylated tau protein. These pathologies lead to progressive and selective neuronal loss in the hippocampus and temporal cortex, cognitive decline and eventual death. There is no curative treatment available for AD at present and current treatments only target the management of symptoms with no influence on disease progression. The pathogenesis of AD has been postulated to be due to the accumulation of Aβ as a result of altered amyloid precursor protein (APP), accumulation of tau, oxidative stress caused by mitochondrial dysfunction and persistent neuroinflammation.

### Neuroprotective effects of E2 in AD

Neuroprotective effects of E2 have been proposed in experimental models of AD. Estrogen deficiency in the brain accelerates Aβ plaque formation (37–39), while E2 treatment has been shown to reduce the expression of Aβ peptide and abnormal accumulation of amyloid proteins (40–42). The reduction of Aβ following E2 administration might be linked to the alteration of the APP gene, as APP protein levels are reduced following E2 treatment (43) as well as the cleavage of APP into toxic Aβ. E2 stimulation increases the secreted APPα, which can lead to a decrease in toxic Aβ species (44, 45). This neuroprotection against β-amyloid toxicity have been shown to occur *via* ERα and ERβ (46). In addition, to protection against Aβ accumulation, E2 is known to also decrease tau hyperphosphorylation in experimental models of AD (47, 48).

A loss of cholinergic neurons is recognized as one of the hallmarks of AD. There is considerable evidence showing the effects of E2 on plasticity and protection of cholinergic neurons through an ERα dependent pathway (49, 50). Accordingly, E2 has been reported to upregulate fiber density of the remaining cholinergic neurons after an excitotoxic insult *via* the mitogen-activated protein kinase (MAPK) signaling pathway, leading to the stimulation of CREB phosphorylation (8, 35, 51). E2 has also been known to alter the dynamics of neural circuits, such as modulating the plasticity of dendritic spines and stimulating neurogenesis and synaptic contacts in numerous brain regions like the hippocampus, hypothalamus and amygdala (52–54). In experimental models of AD, such as the transgenic APP/PS1 and 3xTg AD mice, ovariectomy increased the accumulation of the Aβ peptide and decreased hippocampal-dependent behavioral performance. Treatment with E2 not only prevented the worsening of pathologies, but also reduced the accumulation of Aβ in the hippocampus, subiculum and amygdala (55, 56), suggesting a protective role of E2 in AD progression. With the potential impact of E2 on systemic tissues, there is a need to develop brain-specific therapies. Treatment with a brain-selective prodrug, DHED (10β,17β-dihydroxyestra-1,4-dien-3-one), in APP/PS1 double transgenic mice showed no systemic off-target effects in the uterine tissue, but similar improvements in APP levels, suggesting that the brain-selective treatment with DHED can be used as an early-stage intervention for AD (57).

Taken together, E2 has the potential to regenerate, restore and strengthen the formation of new synaptic networks from the remaining neurons and/or rewire neural circuits under pathological conditions.

### Targeting non-classical E2 pathway as potential treatment in AD

Given the neuroprotective potential of E2 in AD, targeting the non-classical E2 pathway selectively may provide an alternative treatment strategy. Studies have shown that ANGELS compounds, such as estren, can activate the non-classical E2 pathway and rescue the survival of basal forebrain cholinergic neurons after injection of Aβ (1–42) in mice (36) and is neuroprotective against Aβ-induced injury *in vitro* (58). A key important feature of estren treatment is that, unlike E2, it does not increase the size of the uterus, indicating that it might not have unwanted, genomic side effects (59). Regarding cognition, E2 has consistently been reported to have the ability to enhance cognitive function *via* the non-classical E2 pathway involving the ERK1/2 and Akt signaling pathways (60–64). A number of clinical trials in AD have been conducted with the second generation SERM, raloxifene, with varying results, in hope of alleviating cognitive deficits. While some showed that raloxifene improved verbal memory and reduced the risk of AD and mild cognitive impairment, others showed no significant changes in cognition (65–67).

More recent studies show that targeting non-nuclear ERs, such as GPER1, or using non-classical ligands, such as STX, could ameliorate memory impairments or protect against Aβ-toxicity in experimental models of AD *via* activation of the ERK and PI3K/Akt signaling pathways (68–70). These studies provide evidence that activation of the membrane-bound, non-nuclear ERs can provide an alternative therapeutic target in AD. Another novel compound that is of emerging interest is the Pathway Preferential Estrogen-1 (PaPE-1), which is a selective non-nuclear ER pathway activator, which can protect neurons against Aβ-induced toxicity through a mechanism that involves inhibition of oxidative stress and apoptosis (71). This compound strongly activates the MAPK and mTOR pathways without interaction with the nuclear receptors and has a broad spectrum of utility in other neurological disorders, where it also decreases the severity of stroke (72). However, there is a clear lack of clinical trials for these newly developed compounds and more studies are warranted to determine the viability of using non-classical E2 activators as a preventive treatment alternative for AD.

## Parkinson’s disease

### Pathophysiology

Parkinson’s disease (PD) is one of the most common age-related neurodegenerative movement disorders. The main pathological hallmark of PD is motor symptoms consisting of resting tremor, rigidity, bradykinesia and postural imbalance, attributed primarily to the substantial loss of midbrain dopamine (DA) neurons in the substantia nigra pars compacta and the accumulation of α-synuclein cytoplasmic protein deposits, termed Lewy Bodies, in the surviving neurons. The dopaminergic system is not the only affected network in PD. Degeneration of serotonergic neurons in the raphe nucleus, noradrenergic neurons of the locus coeruleus and cholinergic neurons of the nucleus basalis of Meynert have also been reported in PD. Numerous different treatment methods have been investigated to alleviate motor deficits, but no effective clinical therapy has been found to be able to prevent or reverse the degeneration of DA neurons (73). There is currently no cure for PD and available treatments are only symptomatic. DA itself is not a suitable drug as it does not cross the blood-brain-barrier, has a short half-life and has peripheral hemodynamic side effects. Oral administration of L-DOPA remains the gold standard treatment today (74, 75). However, the challenge with L-DOPA is that it cannot be utilized as a long-term treatment for PD. As such, the development of new therapeutics and strategies with several mechanisms of action, such as neurosteroids, could provide an alternative treatment for PD.

### Neuroprotective effects of E2 in PD

While E2 effects on the dopaminergic system have not been well characterized, there is some evidence of a modulatory effect of E2 in PD patients. Postmenopausal women who received hormone replacement therapy have a reduced risk of developing PD and lower disease severity in early stages of the disease (76, 77). E2 has been reported to be protective against 6-OHDA (6-hydroxy dopamine) toxicity in DA neurons (78). Similarly, in the neurotoxin MPTP (1-methyl-4-phenyl-1,2,3,6-tetrahydropyridine) model of PD, E2 treatment improved DA release in the striatum and nucleus accumbens and could protect DA neurons (79–82). In fact, E2 treatment has been shown to increase fiber density of tyrosine hydroxylase-positive DA neurons in both 6-OHDA and MPTP-induced models (83–85). In order to determine the ER subtype regulating neuroprotection in PD, studies have used selective ER agonists and found that the activation of ERα but not ERβ rescued the depletion of DA and prevented the loss of DA transporter in the striatum and cell death in the substantia nigra in MPTP-treated mice (86–88). These studies suggest that neuroprotection of DA neurons occurs through an ERα-specific manner in experimental models of PD.

### Targeting non-classical E2 pathway as potential treatment in PD

There is a lack of research on SERMs in human studies of PD. The majority of the studies have been performed in rodent models with contradictory results. In the MPTP model, raloxifene treatment prevented the MPTP-induced DA depletion, restored DA levels and prevented DA cell death (89, 90) while in other studies was proven ineffective (91). The varying results could be due to differences in the models used, dosing paradigm or pharmacological properties of the different compounds. The other new estrogen analogue, the brain-selective estrogen prodrug, DHED, was found to protect DA neurons in the MPTP-toxicity model and in 3K α-synuclein transgenic mice (mouse model that exhibits many features of PD neuropathology) (92, 93). DHED was also found to selectively increase E2 in the brain while the periphery was spared, which in turn, reduced the secondary effects of E2 on the body (94). In addition, DHED treatment significantly alleviated the neuronal pathology of PD *via* decreasing α-synuclein monomer accumulation and aggregation, restoring vesicle and dopaminergic fiber densities as well as improving PD-associated motor deficits (92–94). Taken together, this evidence highlights the potential for modulating E2 signaling with pharmaceutical analogues for neuroprotection in PD. More investigations into the use of these non-classical activator compounds in PD models are warranted to determine their therapeutic potential.

## Brain injury disorders

### Pathophysiology

Brain injuries can be classified into two main categories, traumatic and non-traumatic. Traumatic brain injury (TBI) occurs when the original function of the brain or the underlying anatomy changes due to an external force (e.g., injury). Non-traumatic brain injury, also referred to as acquired brain injury, is caused by internal factors such as lack of oxygen, exposure to toxins or infection. Examples of non-traumatic brain injury include stroke and cerebral ischemia. Although brain injury is not a neurodegenerative disease per se, it is now clear that brain injuries can trigger progressive neurodegeneration and dementia (e.g., AD) (95). As TBI and stroke are recognized as one of the leading causes of disability and death in most societies (96, 97), it is important to discuss the potential of using alternative non-surgical therapies.

### Neuroprotective effects of E2 in brain injury disorders

The evidence is not clear, especially when it comes to human studies, but there is a strong indication that there is a trend for sex differences, potentially due to differing circulating E2 levels, in the incidence and mortality rate of TBI (98–100). Another indication that E2 might play a role in ameliorating neuronal damage following injury is that the activity of aromatase (a key enzyme in E2 synthesis) increases, particularly in brain astroglia cells (101). This increased aromatase activity has been reported to be neuroprotective in various animal models (102). Besides locally produced E2 in the brain, exogenous E2 application before or immediately after injury has also been shown to rescue damage following a controlled impact in ovariectomized mice (103, 104), indicating that E2 does have treatment potential following trauma in both the TBI and stroke experimental models.

### Targeting non-classical E2 pathway as potential treatment in brain injury disorders

As in the case of other forms of neuronal brain damage, the non-classical estrogen pathway has been reported to have treatment potential in TBI and also in stroke. A known characteristic of TBI is that the primary injury due to the external force is often followed by a slower secondary injury. One of the most common secondary injuries is excessive glutamate release, which is followed by overactivation of NMDA (N-methyl-D-aspartate) and AMPA (α-amino-3-hydroxy-5-methyl-4-isoxazolepropionic acid) receptors and consequentially intracellular ion imbalance, leading to excitatory cell death (105). In an experimental model of NMDA-induced toxicity, E2 treatment following injury ameliorated the damage in basal forebrain cholinergic fibers in mice (35). Importantly, this study highlighted the involvement of the non-classical E2 pathway *via* the MAPK/PKA signaling system. The non-classical pathway activator, estren (a member of the ANGELS compounds), has also been able to trigger E2-like restorative actions. And, as for the receptor dependence of the protective actions of E2 in TBI, the above-mentioned study highlighted that ERα is required for the ameliorative effects after damage (35). However, another study has shown that both ERα and ERβ helped to reduce brain edema following TBI in rats (106). It has also been shown that E2 treatment following TBI can increase ERα and restore ERβ expression in the brain (107). In addition to these classical E2 receptors, it appears that GPER1 is also involved in neuroprotection following TBI. Both E2 and treatment with the GPER1 agonist, G-1, increased neuronal survival as well as decreased neuronal degeneration and apoptotic cell death in a rodent model of TBI (108). These results were corroborated in other rat TBI studies, where G-1 was found to promote neuronal survival and improve cognitive impairment (109) as well as reduced neuronal apoptosis and increased microglia polarization (110), through the PI3K/Akt signaling pathway. Likewise, the non-classical pathway has also been implicated as an alternative treatment in other brain injury disorders. Treatment with G-1 improved neuronal survival after brain ischemia, reduced infarct size, neuronal injury and improved neuroinflammation and immunosuppression after experimentally induced stroke and cerebral ischemia (104, 111, 112). Furthermore, treatment with other non-classical pathway activators, such as PaPE-1 and the SERM bazedoxifene, protected neurons against ischemic brain damage in rodents and in neuronal culture, potentially through the MAPK/ERK1/2 signaling pathway (113, 114).

Neuroinflammation can play a key role in the secondary injury observed in TBI as well as after stroke with the activation of microglia cells, among others, and the release of inflammatory factors (115–117). Following TBI, G-1 exerts anti-inflammatory effects, but it appears that there are sex specific differences as these results were observed in males and ovariectomized females, but not in intact females. Therefore, the circulating levels of E2 in patients will likely influence any potential medical treatment following brain injury. In addition to G-1, STX has also been found to be capable of attenuating ischemia-induced neuronal loss in middle-aged rats (30). Importantly, this study showed that animals which have not been exposed to E2 for some time still maintained their responsiveness to E2 and E2-like compounds as treatment, highlighting the use of non-feminizing estrogens, that can be candidates in both males and females and at different age groups. Taken together, these results strongly suggest that the non-classical pathway can be targeted as potential treatment in traumatic and non-traumatic brain injury disorders.

## Conclusions

In this mini-review, we discussed the neuroprotective role of E2 and the potential involvement of the non-classical estrogen pathway in ameliorating or alleviating disease phenotype in experimental models of AD, PD and brain injury disorders. The results from *in vivo* and *in vitro* studies with selective non-classical pathway activators, such as raloxifene, estren, STX, G-1, PaPE-1 and DHED, are very promising targets and present hopeful beneficial effects on their potential use as treatment in neurodegenerative diseases. However, as both the classical and non-classical pathways are intact in most, if not all, of these studies, it is difficult to ascertain whether the observed neuroprotective effects of E2 are solely attributed to the non-classical pathway. Some of the ongoing challenges with these selective non-classical pathway activators include how to modulate selectivity and sensitivity to ensure that the non-classical pathway is stimulated without triggering the classical pathway. Extra caution also needs to be taken in their interpretation as, at present, there is a lack of conclusive evidence for their use in the human brain. More studies are warranted to translate these neuroprotective effects in human clinical trials before they can be utilized as a novel therapeutic strategy to ameliorate, prevent the onset and/or slow down disease progression in neurodegenerative diseases.

## Author contributions

Both ZK and RC developed the concept and wrote the manuscript. Both authors have made a substantial, direct and intellectual contribution to the work and approved the manuscript prior to its submission.

## Conflict of interest

RC was employed by Timeline Bioresearch AB.

The remaining author declares that the research was conducted in the absence of any commercial or financial relationships that could be construed as a potential conflict of interest.

## Publisher’s note

All claims expressed in this article are solely those of the authors and do not necessarily represent those of their affiliated organizations, or those of the publisher, the editors and the reviewers. Any product that may be evaluated in this article, or claim that may be made by its manufacturer, is not guaranteed or endorsed by the publisher.
